# Unlocking microalgal host—exploring dark-growing microalgae transformation for sustainable high-value phytochemical production

**DOI:** 10.3389/fbioe.2023.1296216

**Published:** 2023-11-09

**Authors:** Surumpa Jareonsin, Kanjana Mahanil, Kittiya Phinyo, Sirasit Srinuanpan, Jeeraporn Pekkoh, Masafumi Kameya, Hiroyuki Arai, Masaharu Ishii, Ruttaporn Chundet, Pachara Sattayawat, Chayakorn Pumas

**Affiliations:** ^1^ Department of Biology, Faculty of Science, Chiang Mai University, Chiang Mai, Thailand; ^2^ Applied Microbiology (International Program) in Faculty of Science, Chiang Mai University, Chiang Mai, Thailand; ^3^ Office of Research Administration, Chiang Mai University, Chiang Mai, Thailand; ^4^ Center of Excellence in Microbial Diversity and Sustainable Utilization, Chiang Mai University, Chiang Mai, Thailand; ^5^ Department of Biotechnology, Graduate School of Agricultural and Life Sciences, The University of Tokyo, Bunkyo-ku, Tokyo, Japan; ^6^ Division of Biotechnology, Faculty of Science, Maejo University, Chiangmai, Chiang Mai, Thailand; ^7^ Environmental Science Research Centre, Faculty of Science, Chiang Mai University, Chiang Mai, Thailand; ^8^ Multidisciplinary Research Institute, Chiang Mai University, Chiang Mai, Thailand

**Keywords:** microalgae, genetic engineering, heterotroph, phytochemicals, chlorella, agrobacterium, transformation

## Abstract

Microalgae have emerged as a promising, next-generation sustainable resource with versatile applications, particularly as expression platforms and green cell factories. They possess the ability to overcome the limitations of terrestrial plants, such non-arable land, water scarcity, time-intensive growth, and seasonal changes. However, the heterologous expression of interested genes in microalgae under heterotrophic cultivation (dark mode) remains a niche area within the field of engineering technologies. In this study, the green microalga, *Chlorella sorokiniana* AARL G015 was chosen as a potential candidate due to its remarkable capacity for rapid growth in complete darkness, its ability to utilize diverse carbon sources, and its potential for wastewater treatment in a circular bioeconomy model. The aims of this study were to advance microalgal genetic engineering *via* dark cultivation, thereby positioning the strain as promising dark-host for expressing heterologous genes to produce high-value phytochemicals and ingredients for food and feed. To facilitate comprehensive screening based on resistance, eleven common antibiotics were tested under heterotrophic condition. As the most effective selectable markers for this strain, G418, hygromycin, and streptomycin exhibited growth inhibition rates of 98%, 93%, and 92%, respectively, ensuring robust long-term transgenic growth. Successful transformation was achieved through microalgal cell cocultivation with *Agrobacterium* under complete darkness verified through the expression of green fluorescence protein and β-glucuronidase. In summary, this study pioneers an alternative dark-host microalgal platform, using, *Chlorella*, under dark mode, presenting an easy protocol for heterologous gene transformation for microalgal host, devoid of the need for expensive equipment and light for industrial production. Furthermore, the developed genetic transformation methodology presents a sustainable way for production of high-value nutrients, dietary supplements, nutraceuticals, proteins and pharmaceuticals using heterotrophic microalgae as an innovative host system.

## 1 Introduction

Is traditional production both sustainable and sufficient? The current need for sustainable platforms, resources, and cell factories, particularly for the production of valuable phytochemicals has prominently directed attention towards microalgal production due to its short doubling time. This focus is driven by its potential alignment with the concept of a bio-circular economy model, which aims to make the most of resources. This microalgal production and applications sector have undergone a significant shift over the past 5 years (Maria et al., 2023). With the global population projected to surge to 9.9 billion by 2050 (United Nations, 2023), coupled with severely environmental shifts, the advancement of biotechnologies has become important to solve these issues. Utilizing alternative green cell factories—specifically microalgae—to express foreign genes can enhance foods, feeds, recombinant proteins, biopharmaceuticals, and high-value compounds.

A wide range of biological chemicals is derived from fruits, vegetables, whole grains, and other part of plants. These compounds produced by plants are known as phytochemicals. However, concerns regarding plant cultivation limitations persist, such as arable land, seasonal changes, climatic conditions, time-consuming processes, and high production costs (Jareonsin and Pumas, 2021). In order to address these challenges, a sustainable approach to develop eco-friendly industries has been required. Most biopharmaceutical products are currently manufactured in animal cells, but each host has its limitations, including low yield, high cost, virus contamination, and expensive medium costs, among others. As a result, alternative hosts for phytochemical production have been continually investigated. In this sense, eukaryotic microalgae hold high metabolic potential within the context of the circular bioeconomy concept, and offer tremendous metabolic potential to serve as a suitable platform for plant chemicals production, as mentioned earlier. Genetic engineering of microalgae provides cutting-edge tools to expand the platform for food and feed production (Kusmayadi et al., 2021). Furthermore, eukaryotic algae not only share evolutionary ancestry with land plants but also fulfill most of the necessary criteria, particularly for plant chemicals (Novoveska et al., 2019; Saini et al., 2019). They exhibit metabolic potential and post-translational modification pathways appropriate for phytochemical production (Weiner et al., 2018). Additionally, microalgal metabolism precursors are more closely associated with phytochemicals compared to those in prokaryotic hosts (Lauersen et al., 2018). Therefore, further developments are needed to establish microalgae as a regular resource for interested compounds.

Microalgal genera such as *Arthrospira*, *Dunnaliella*, and *Chlorella* have emerged as an ideal platform for large-scale production due to their Generally Recognized as Safe (GRAS) status recognized by the Food and Drug Administration (FDA) (Yaakob et al., 2014). Among these, *Chlorella* holds significant industrial potential due to several key factors, including high growth rate, ability to grow in mass culture reactors over extended periods, robustness in coping with various harsh conditions. With an expected value of USD 412.3 million by 2028, *Chlorella* sp. holds one of the highest market values, with a targeted yield of 5,000 tons dry matter per year (Levasseur et al., 2020). Additionally, *Chlorella* is considered to be safe with lower risk of viral, prion, or bacterial endotoxin contamination (Yaakob et al., 2014). Moreover, *Chlorella* exhibits high nutritional value and high lipid content exceeding that of most terrestrial plants and is recognized as a source for biofuel production. Thus, recombinant products derived from microalgae hold significant advantages and are closely related to secondary metabolites produced by desired plant genes. Currently, plant-derived ingredients have shown positive correlations in numerous plant ingredients aspects. These include the utilization of phytochemical ingredients produced from microalgae as a manufacturing, such as polyunsaturated fatty acid (PUFAs) for nutritional purposes, cannabinoids for medical use, terpenoids for pigments and supplements, Cytochrome P450s for plant metabolites, astaxanthin for food coloring, hydrocarbon for high-quality fuel applications, and plant hormone (Laban, 2019; Jareonsin and Pumas, 2021).

With the advancements in microalgal biotechnologies, including genomics, bioinformatics, analyses, and genetic and metabolic engineering, further studies should focus on developing heterotrophic hosts for novel products synthesis through gene insertion. Microalgae offer sustainability advantages as an alternative expression host for recombinant production due to their ability to perform correct post-transcriptional and post-translational modifications, cost-effectiveness, and shorter expression times without significant land or water usage (Yang et al., 2016). However, the field of microalgal engineering is still relatively new, and the engineering technologies for microalgae are not as well-developed as those for heterotrophic microbes (Lu et al., 2021). Microalgal biotechnology currently faces several challenges as follow: i) limited model microalgal species, ii) limited reports on expression and transformation under heterotrophic mode, iii) low transformation efficiencies, and v) despite the greater advantages offered by heterotrophic microalgae compared to autotrophic microalgae, there is still a lack of knowledge regarding the biocircular economic model for utilizing sustainable organisms as a dark host for future industrial development. The benefits of dark microalgal hosts include more economical nutrient options, lower instrument costs, and ease of operation and maintenance (Jareonsin and Pumas, 2021). Their rapid growth can adapt to large-scale production within a few weeks without concerns about light penetration, leading to reduce costs related to employee hiring time, and electricity when using artificial light sources, and so on (Yang et al., 2016).

**FIGURE 1:**
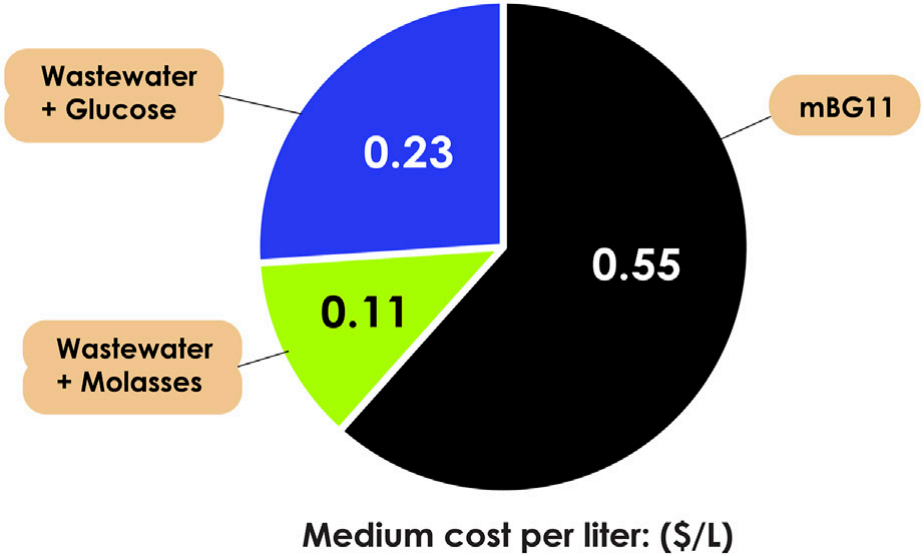
Cost analysis of *Chorella sorokiniana* AARL G015 using commonly used medium (mBG11) and wastewater with different organic carbon sources (glucose and molasses).

In heterotrophic microalgal cultivation, 80% of the production cost depends on organic carbons sources (Park and Moon, 2018), while bacterial cultivation can reach up to 60% (Omoregie et al., 2019). Certain heterotrophic microalgae, particularly *Chlorella*, can be cultivated using wastewater or by-products as nutrient sources, contributing to both economic and environmental sustainability (EI-Sheekh et al., 2014). For example, in our previous studies (Chen et al., 2019; Jareonsin et al., 2023), we found that the most cost-effective option is to use poultry effluent with molasses as carbon sources, saving up to 80% compared to mBG11 medium (reducing the cost from $0.55 to $0.11) and 52% compared to the wastewater with 10 g/L glucose (reducing the cost from $0.23 to $0.11), in term of cost, as shown in Figure 1. While bacteria cultivation with commonly used media such as yeast extract, nutrient broth, cooked meat medium, and lactose broth can cost $3.56, $1.59, $134.06, and $2.19 per liter (Omoregie et al., 2019). Utilizing wastewater or by-product strategies for heterotrophic microalgal hosts offers an alternative method compared to employing other heterotrophic host organisms like bacteria and yeast. Even though bacteria can use inexpensive media, it is crucial to note that certain low-cost media may lack rigorous quality control and reproducibility, especially in ureolytic bacteria (Cuzman et al., 2015). The price to be paid not only the production costs, but also the following effects from many dimensional disadvantages in terms of applicability, environmental impacts, and cost-effectiveness. In another perspective, microalgae hosts can indirectly contribute to the reduction of their entire production chain. For instance, in certain countries, like Spain, cost can exceed $1.72/kg for various chemicals and $0.13/kWh for energy use in cleaning industrial wastewater (Ruiz et al., 2022).

Additionally, yeast*, Saccharomyces cerevisiae*, cultivated on laboratory medium costs $0.85 per liter, while using batch production medium can cost $0.035-$0.045 per liter (Malairuang et al., 2020). In this sense, coculture with yeast and microalgae could reduce the cost and enhance the interested product (Qin et al., 2019). For example, the co-cultivation of *Chlorella* sp. and *Rhodotorula glutinis* in starch processing effluent has the capability to more efficiently convert nutrients into single-cell compared to the monoculture approach. Consequently, the co-fermentation field of microalgae and yeast emerges as a promising and practical strategy for cost-effective and sustainable production (Lu et al., 2023). Furthermore, an effective and industrially scalable heterotrophic *Chlorella sorokiniana* GT-1 could reduce costs to $1,601 per ton of biomass if the biomass concentration reached 200 g/L, spending annual costs of equipment depreciation and power consumption lower than *Chlorella protothecoids*, reducing 24% of the overall production cost (Jin et al., 2021). In a particular gene aimed to produce plant substances for future phytochemical applications, microalgae may offer more favorable conditions for genetic engineering compared to yeast. Moreover, microalgae metabolism involves the production of precursors more closely associated with phytochemicals than that of prokaryotic hosts (Lauersen et al., 2018). Despite yeast is an excellent eukaryotic host due to its low cost a scalability, the common occurrence of hypermannosylation in yeast can result in misfolded proteins and activity malfunction (Yusibov and Mamedov, 2010). This occurrence may result in extra costs depending on the genes of interest.

However, a limited number of large companies, such as Terravia Holding, Inc., Nutrinova, DSM, and Corbion are engaged in heterotrophic cultivation (Santin et al., 2022). While autotrophic cultivation is the main strategy in microalgal production, there are some limitations (Ruiz et al., 2022). New insights are needed for this field to enhance transformation efficiencies, establish specific transformation systems for individual strains and products, develop target editing methods, explore alternative advance technologies for microalgae, and address the remaining challenges, particularly by using microalgae that can easily and completely grow under dark cultivation as a heterotrophic host.

Is it possible to grow microalgae without using light? Most microalgae are capable of growing in wide range of environments. The three cultivation modes for microalgae—autotrophic, mixotrophic, and heterotrophic cultivation—are categorized by the source of energy and carbon (Dreesen et al., 2010). As the economic and industrial scales increase to meet human demands, heterotrophic cultivation (dark mode) holds a significant advantage in biomass production and economic value in larger scale, especially for high-volume and cost-effectiveness. Heterotrophic cultivation enables the utilization of wastewater, offers easy control of factors, supports bioremediation, and operates within a closed system that requires less environmental factors compared to autotrophic and mixotrophic cultivation, which rely on light for being the source of energy (Chen et al., 2023).

In the search of a suitable microalga strain to develop innovative biotechnology, the green microalga, *Chlorella sorokiniana* has intrigued as a candidate due to its enriched fast-growing nature and its potential for multiple applications across various industries under different cultivation modes (Park et al., 2019; Yun et al., 2020; Politaeva et al., 2021). Our previous studies have revealed that *Chlorella sorokiniana* AARL G015 exhibits robust growth, reaching full nutrient utilization within a short period of 5–14 days, and achieving higher biomass production (3–6 g/L) in dark mode (Jareonsin et al., 2023). This cultivation mode reduces the cost of production as low as 0.02 $/g. As recombinant microalgae strains are being developed, it is crucial to evaluate the expression of heterologous genes under various cultivation types, particularly in dark cultivation as mentioned above. Fortunately, some of the most common microalgae are capable of heterotrophic growth. However, there is a lack of researches in the engineering field and further investigations are required. Heterotrophic microalgal host, referred to as a ‘dark host’ in this context, can be independently managed without the risk of environmental contamination, as they can be cultivated in a fermenter or closed system (Khan et al., 2016). However, information on transgene expression under dark mode with *C. sorokiniana* for strain development is very limited and needed for studies and developments.

*Agrobacterium tumefaciens*-mediated transformation technique is one of the methods used to deliver genes into host cells. This technique has shown success in a broad spectrum of cells, especially from both plant and microalgae, such as *Chlamydomonas reinhardtii*, *Chlorella vulgaris*, *Dunaliella salina*, *Haematococcus pluvialis*, and others (Dehghani et al., 2018). Furthermore, a new assay for the screening of transgenic strains has been developed which employs quantitative analysis of β-glucuronidase (GUS) histochemical assay with X-gluc (Yedahalli et al., 2018). Based on other studies, the transformation efficiency of three microalgal species was found to be as follows: *Chlorella* sp (12.25%), *Scenedesmus bajacalifornicus* (2.92%) and *Ankistrodesmus* sp. (3.5%) using *Agrobacterium* transformation method (Sanitha et al., 2014). This transformation method is flexible and easy to perform with less cost; however, there are limited research studies providing specific results for microalgal strains. Therefore, there is a gap to fill for more accessible knowledge and information regarding heterologous expression in microalgae.

Therefore, there is a need to identify a new eukaryotic microalgal dark host that can effectively produce heterologous proteins and phytochemicals for various biotechnological applications to improve quality of nutrition. In this study, the genetic modification of a specific dark microalgal host was evaluated as a promising alternative host. Our aim was to produce phytochemicals, and develop an efficient transformation system for *Chlorella* as a dark host, thereby enhancing the potential of transgenic microalgae for the production of desirable substances to meet human needs. Up to our knowledge, this is the first report of a genetic engineering system in *C. sorokiniana* under completely dark cultivation *via Agrobacterium*-mediated transformation. The increasing interest in finding new and innovative algal host systems and platforms is motivated by the potential to create sustainable organisms that can serve as hosts or cell factories. This approach aims to address the challenges related to the requirement for light, cost, and energy at an industrial scale.

## 2 Materials and methods

### 2.1 Heterotrophic culture maintenance

*Chlorella sorokiniana* AARL G015 (GenBank accession number OR234725) was obtained from a septic tank at the Biogas Plant Chicken farm in Lamphun district, located in the north of Thailand. This strain was accidently isolated using actinomycetes ISP2 medium (Kumsiri et al., 2021). According to our previous study (Jareonsin et al., 2023), this strain showed faster growth and higher biomass production under heterotrophic mode in modified Blue Green Medium (mBG11). The composition of mBG11 included the following components per g/L: NaNO_3_ 1.5, K_2_HPO_4_ 0.04, MgSO_4_.7H_2_O 0.075, CaCl_2_.2H_2_O 0.036, citric acid 0.006, ammonium ferric citrate green 0.006, EDTANa_2_ 0.001, Na_2_CO_3_ 0.02, glucose 10, and yeast extract 2. The trace metal solution consisted of H_3_BO_3_ 2.86, MnCl_2_.4H_2_O 1.81, ZnSO_4_.7H_2_O 0.22, Na_2_MoO_4_.2H_2_O 0.39, CuSO4.5H_2_O 0.08, and Co. (NO_3_)_2_.6H_2_O 0.05. (Kuhl and Lorenzen, 1964). The seed cultures were plated on mBG11 and incubated at 25°C. The culture was maintained aseptically in a dark room at temperature of 25°C ± 1°C.

### 2.2 Cell growth under dark mode

The cell cultures were initiated using an inoculum cell density of 1 × 10^6^ cells/mL in the exponential phase, with a total volume of 50 mL of mBG11. The culture conditions were maintained in heterotrophic mode with shaking at 180–200 rpm. Cell density was measured for 2 weeks using a hemocytometer (Neubauer Improved Bright-Line, HBG Germany). Growth parameters, specific growth rate (µ) and doubling time (dt) were calculated.

Gravimetry was measured by dry cell weight of biomass per volume (DCW). A 10-mL of the strain culture was filtered through microfiber filters (Whatman GF/C, 47 nm m diameter), dried at 60°C for 24–48 h, cooled under vacuum 30 min and weighed (Lin et al., 2012). The cell dry weight was measured every day for 7 days, and it was calculated using Eq. 1

DCW=Wm−V (1)

In these equations Wm represents the net weight of membrane before and after filtration, and V represents the filtration volume.

### 2.3 Plasmid construction and bacterial strains

The binary vector pCAMBIA1304 (ABCAM, United Kingdom), which contained β-glucuronidase (*gus A*) and green fluorescent protein (*mgfp5*) as reporters driven by the CaMV 35S promoter, was used for transformation. This vector has been employed across a wide range of plant and green microalgal species, including *Chlamydomonas* (Cha et al., 2012). The specific sequence in the pCAMBIA vector responsible for T-DNA integration is the T-DNA borders, comprising of the Left Border (LB) and Right border (RB) (Gelvin, 2021). These borders contain specific recognition sites for the *Agrobacterium* VirD1 and VirD2 proteins, essential for the identification, processing, and subsequent integration of T-DNA into the plant genome by *Agrobacterium*.

Hygromycin resistance was used to select transformed host cells as the binary vector carried the selectable marker, hygromycin phosphotransferase II (hpt II) gene. The binary vector was introduced into *A. tumefaciens* strain LBA4404 through a heat shock procedure, following the method described by Graciano et al. (2018). The *A. tumefaciens* strains carrying pCAMBIA1304 were maintained on LB agar plate supplemented with rifampicin (20 μg/mL) and kanamycin (50 μg/mL).

### 2.4 Selectable markers: resistant antibiotics in dark mode

To assess a potential effect of antibiotics on *C. sorokiniana* AARL G015 in heterotrophic cultivation, the microalga was screened for sensitivity to 11 antibiotics, which are common selectable markers; kanamycin (KAN), streptomycin (STR), ampicillin (AMP), chloramphenicol (CLO), spectinomycin (SPEC), hygromycin (HYG), neomycin (NEO), zeocin (ZEO), G418, rifampicin (RIF), and cefotaxime (CTX). The antibiotic sensitivity was repeatedly determined by inoculating an exponential-phase of 5 × 10^8^ cells/mL in liquid mBG11 with varying concentrations of antibiotics (0, 25, 50, 100, 250, 500, 1,000 μg/mL), using a non-antibiotic culture as a control as in Table 1. Additional experiments were conducted for the antibiotics RIF and CTX, with higher concentrations at 0, 250, 500, 1,000 μg/mL. The culture was grown under dark mode for 14 days, as described previously, with an initial cell density of approximately 5 × 10^8^ cells/mL, and each antibiotic test was repeated at least three times. The highest inhibitory concentration of antibiotics was used as the selection medium and vector construction for further transformation studies. Growth data were measured by cell count and optical density (OD_680_) every 2 days. The percentage of inhibited growth (I%) was evaluated using Eq. 2; where A represents the absorbance at 680 nm.

$$I\%=\frac{\left(Acontrol-Atreatment\right)}{Acontrol}x\;100\%$$ (2)

**TABLE 1:** Lower/higher batch of different concentrations of antibiotics under dark mode.

No.	Antibiotics	Lower concentration	Higher concentration
		(µg/mL)	(µg/mL)
**1**	Kanamycin (KAN)	25, 50, 100	250, 500, 1000
**2**	Ampicillin (AMP)	25, 50, 100	250, 500, 1000
**3**	Hygromycin (HYG)	25, 50, 100	250, 500, 1000
**4**	Streptomycin (STR)	25, 50, 100	250, 500, 1000
**5**	Chloramphenicol (CLO)	25, 50, 100	250, 500, 1000
**6**	Spectinomycin (SPEC)	25, 50, 100	250, 500, 1000
**7**	Neomycin (NEO)	25, 50, 100	250, 500, 1000
**8**	Zeocin (ZEO)	25, 50, 100	250, 500, 1000
**9**	G418	25, 50, 100	250, 500, 1000
**10**	Cefotaxime (CTX)	-	250, 500, 1000
**11**	Rifampicin (RIF)	-	250, 500, 1000

### 2.5 *Agrobacterium*-mediated transformation

**FIGURE 2:**
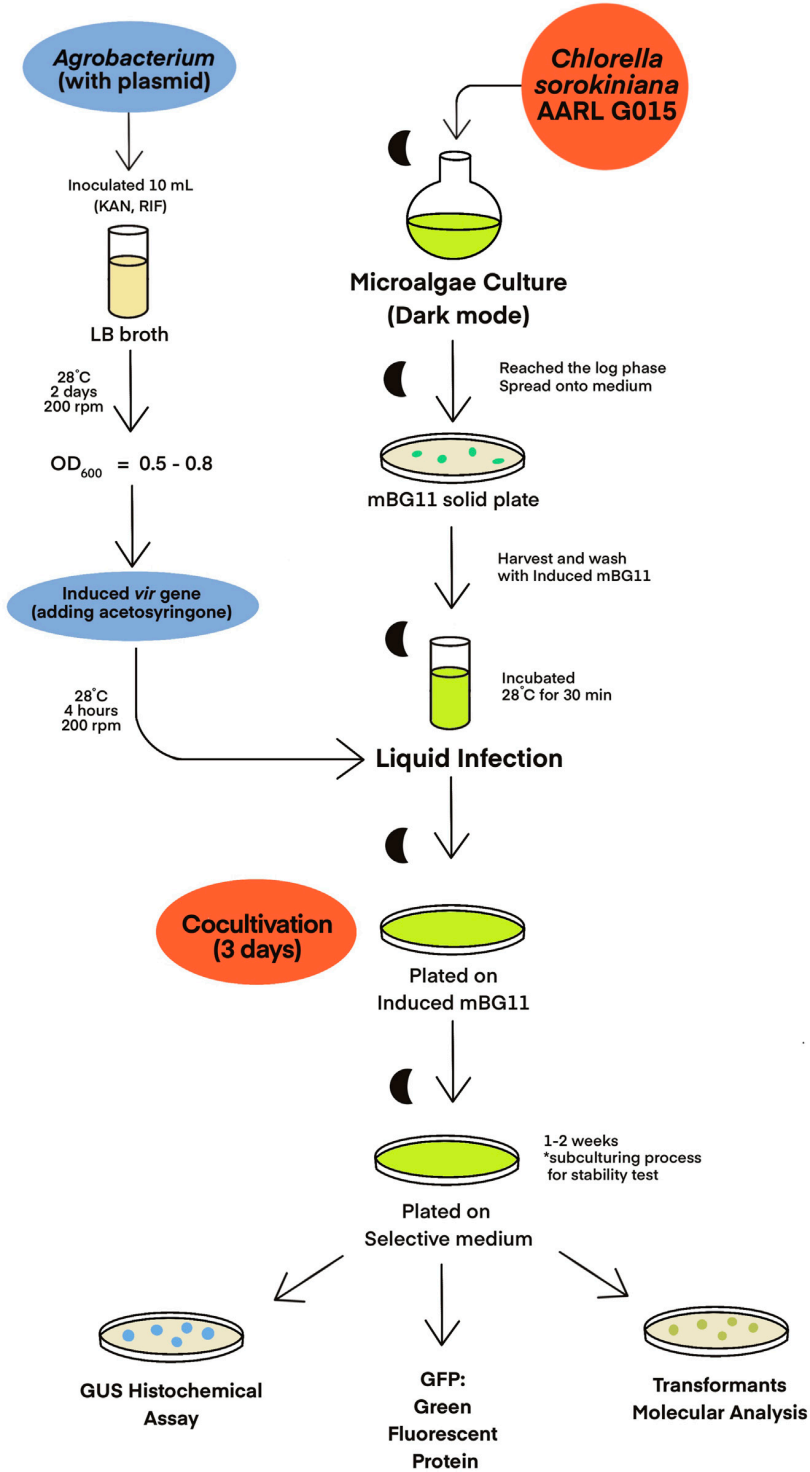
Schematic illustration of *Agrobacterium*-mediated transformation in *Chlorella* sp.

*C. sorokiniana* AARL G015 was transformed by using an *Agrobacterium*-mediated transformation method, which was modified from the protocols described by Cha et al. (2012), Norzagaray-Valenzuela et al. (2018), and Sharma et al. (2021) (Figure 2). For *Agrobacterium* cells, a loopful of cells from the *Agrobacterium* plate carrying the binary vector were inoculated into 10 mL LB medium supplemented with 50 μg/mL kanamycin and 20 μg/mL rifampicin. The culture was incubated at 28°C for 2 days in a shaking incubator at 200 rpm in the dark until OD_600_ reached 0.5–0.8. To induce the virulence (vir) gene, the *Agrobacterium* cells were centrifuged at 4500 *g* for 5 min, and the cell pellets were resuspended in an induced medium (mBG11 + 100 µM acetosyringone). The suspension was then incubated at 28°C for 4 h with shaking 200 rpm.

Prior to cocultivation, the microalgal cultures were initiated from a single colony in 10 mL of mBG11 medium and incubated under the heterotrophic condition. Once the culture reached the exponential phase, the culture was plated onto solid mBG11 medium and allowed to grow into a uniform lawn. On the day of cocultivation through liquid infection, the microalgal cells were harvested and washed twice with an induced medium (mBG11 + 100 µM acetosyringone). The cell pellet was then resuspended in 1,000 µL of *A. tumefaciens* culture which had been grown to OD_600_ of 0.5-1 and incubated at 28°C for 30 min with gentle agitation. The mixture was spread onto a solid plate of the induced medium, and the co-culture was incubated for 3 days at 25°C under dark condition.

After the cocultivation period, the cells were harvested using 7 mL of mBG11 medium containing 500 mg/L cefotaxime to eliminate *Agrobacterium* through heterotrophic growth. Subsequently, the microalgal cells were centrifuged at 4,500 rpm for 1 min and washed twice with deionized water. The cell pellets were plated on selective media, each containing in specific antibiotic and 500 mg/L cefotaxime, and incubated in complete darkness. Resistant colonies began to appear within a week. The resistant clones were regularly transferred to new mBG11 containing the specific antibiotics every 2 weeks. Analysis of GUS and GFP expression in the transformants were detected to confirm successful transformation.

### 2.6 GUS histochemical assay

In order to identify transgenic cells with reporter gene, GUS coding sequence controlled under 35S promoter was monitored. The expression of the reporter was detected using the biochemical GUS assay, as described by Jefferson et al. (1987). The transformants were harvested by centrifugation (6,500 rpm, 5 min, 25°C) and resuspended in a freshly prepared staining buffer containing the X-gluc (5-bromo-4-chloro-3indoyl-β-D-glucuronic Acid; Sigma, St. Louis, MO), then incubated overnight at 37°C in the dark. After incubation, the cells were rinsed with 70% ethanol for 4–5 h to remove chlorophyll. GUS expression was visualized as indigo-blue spots under digital microscope. All experiments were done in triplicates, and the data were analyzed according to the methods described by Sanitha et al. (2014), Dehghani et al. (2018), and Zou et al. (2018).

### 2.7 Stability of the transformants

The transformants were maintained on media without the selectable marker for 2 weeks prior to subculturing onto the selectable media, both with and without antibiotics. This step aimed to assess the persistence of the gene transformation without the presence of selection pressure. The subculturing process was iterated every 2 weeks for a total of 3-5 rounds.

### 2.8 Statistical analysis

All the experiments were done in triplicate treatments. Data analysis was done by using SPSS 10. One-way Analysis of Variance (ANOVA) was used, and Least Significant Differences (LSD) were calculated at significance level of *p* = 0.05 (LSD 0.05).

## 3 Results and discussion

### 3.1 Biomass growth under dark cultivation

*Chlorella sorokiniana* AARL G015 was successfully cultivated in mBG11, which our previous study demonstrated to be the most suitable medium for this strain under heterotrophic cultivation (Jareonsin et al., 2023). Glucose was selected as a carbon source due to its common use in industrial fermentation processes (Shu, 2007). The cell growth revealed the maximum specific growth rate (µ_max_) of 8.52 days^-1^ and doubling time (dt) of 0.14 days. In this medium, the microalga rapidly grow within 7 days after a 4-day lag phase and then last approximately 10–14 days before entering the stationary growth phase. The exponential phase was chosen to perform genetic transformation procedures. The cells obtained through spread and streak plating were inoculated to liquid cultures for transformation.

**FIGURE 3:**
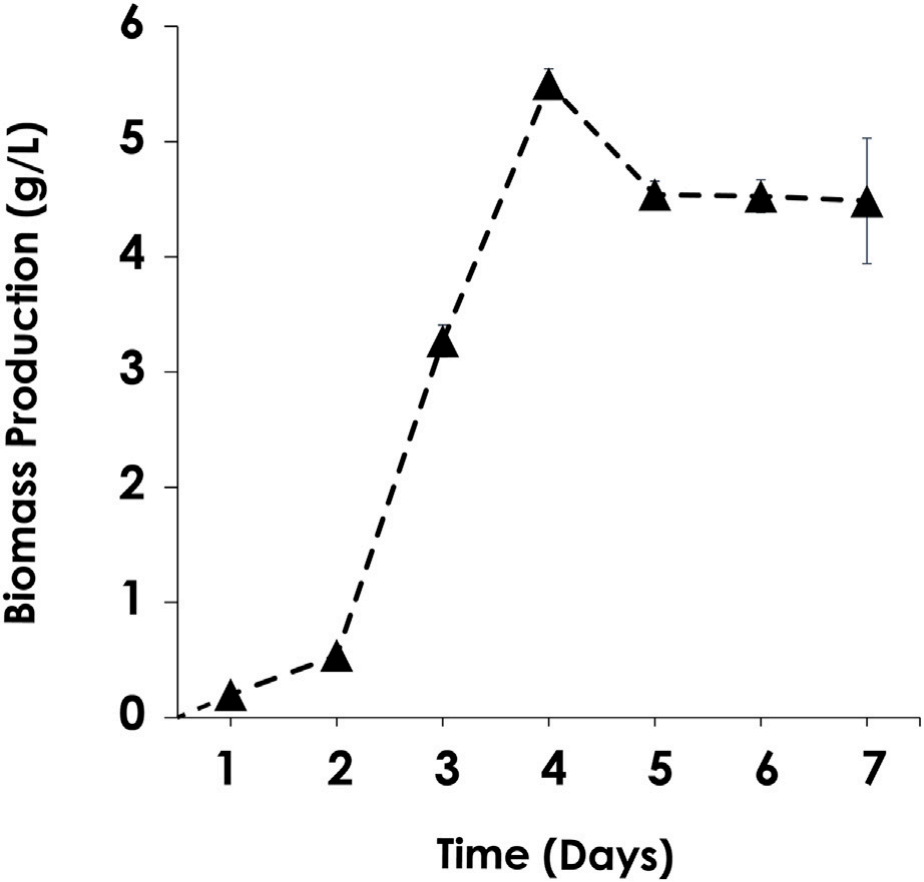
*Chlorella sorokiniana* AARL G015 growth characteristic under heterotrophic cultivation in mBG11.

As illustrated in Figure 3, the biomass dry weight was determined by harvesting microalgal cells for a week. The maximum biomass production of *C. sorokiniana* AARL G015 occurred on the fourth day, reaching 5.5 g/L. This production is 8.6 times higher than the maximum cell yield of *C. vulgaris*–a common model for microalgal host–under heterotrophic condition (Cai et al., 2021). It is also 3.1 times higher than that of *C. sorokiniana* FC6 IITG cultivated under mixotrophic cultivation (Kumar et al., 2014) (Table 2). Subsequently, dry biomass production dropped after 4 days. The cells slightly decreased from fifth to seventh day, after which the cells entered the stationary phase. This result indicated that this strain is suitable for cultivation under dark mode. Moreover, these results implied that this strain holds promise for large-scale industrial applications due to its fast growth and high yield production. Furthermore, once the targeted gene is expressed, this microalga has the potential to produce a substantial of crucial amino acids such as arginine, lysine and cysteines as reported by Chen et al. (2023), which could serve as an alternative protein source for reducing or replacing fish meal in aquafeeds, meeting the urgent demand. Recently, other research has focused on upscaling *C. sorokiniana* FZU60 to achieve ultra-high lutein production under dark mode. The novel fed-batch strategy, as demonstrated by Xie et al. (2022), has shown to significantly enhance the performance and commercial viability of lutein derived from microalgae. After the transformation, the microalgal host can continually be employed under dark cultivation with expression, making this upcoming strain viable for multipurpose. Additionally, understanding the specific growth under specific conditions is crucial before assessing other criteria of host platform. This growth evaluation will contribute to a successful cultivation process, potentially enabling high-density cultures that facilitate cheaper and easier downstream processing steps on a scaling-up (Geada et al., 2023).

**TABLE 2:** Growth characteristics of *Chlorella* sp.

Microalgae	*C. sorokiniana* AARL G015	*C. sorokiniana* FC6 IITG	*C. vulgaris*	*C. vulgaris*
**Medium**	mBG_11_	mBG_11_	Modified Bristol	mBG_11_
**Cultivation mode**	Heterotroph	Mixotroph	Heterotroph	Heterotroph
**Carbon source**	Glucose (10 g/L)	Sodium acetate	Glucose (20 g/L)	Glucose (10 g/L)
**Scale**	50–100 mL	100 mL	200–500 mL	150 mL
**Specific growth (µ, d^−1^)**	5.13	-	1.371	1.18
**Biomass (g/L)**	5.5	1.75	-	0.64
**Biomass productivity (g/L/D)**	2.48	0.11	0.687	3.2
**Lipid content (%)**	11.69	39.2	-	-
**References**	This study	Kumar et al. (2014)	Cao et al. (2023)	Cai et al. (2021)

Other studies support the idea that *Chlorella* can utilize waste more efficiently than *Chlamydomonas* due to its ability to digest and use waste nutrients while simultaneously removing pollutants from wastewater. This implies that in the near future, the use of *Chlorella* in genetic field can also meet the bio-circular model by using waste for culturing. However, the genetic toolbox of *Chlamydomonas* is more advanced than that of *Chlorella*. Therefore, the need to develop approaches across various strains and cultivation modes is crucial.

### 3.2 Selectable marker for dark *Chlorella* host

We investigated the growth response of *C. sorokiniana* AARL G015 to different concentrations of commonly used antibiotics and determined the optimal screening conditions for liquid culture transformed cells. To ensure accurate results and long-term stability, a range of antibiotic concentrations from low to high was tested to verify the percentage of growth inhibition to prevent false positive transformants. Furthermore, for *Agrobacterium* transformation, the elimination of this bacterium after infection is crucial. This is typically achieved using CTX or RIF. Therefore, the antibiotic sensitivity of CTX and RIF was tested at high concentrations (250, 500, 1,000 μg/mL) to confirm that these two antibiotics have less effects on microalgal growth. This evaluation ensures that in *Agrobacterium*-mediated transformation, which often involves the use of these two antibiotics in protocols for bacteria elimination, there is no significant interference with the growth of transgenic microalgal cells.

**FIGURE 4:**
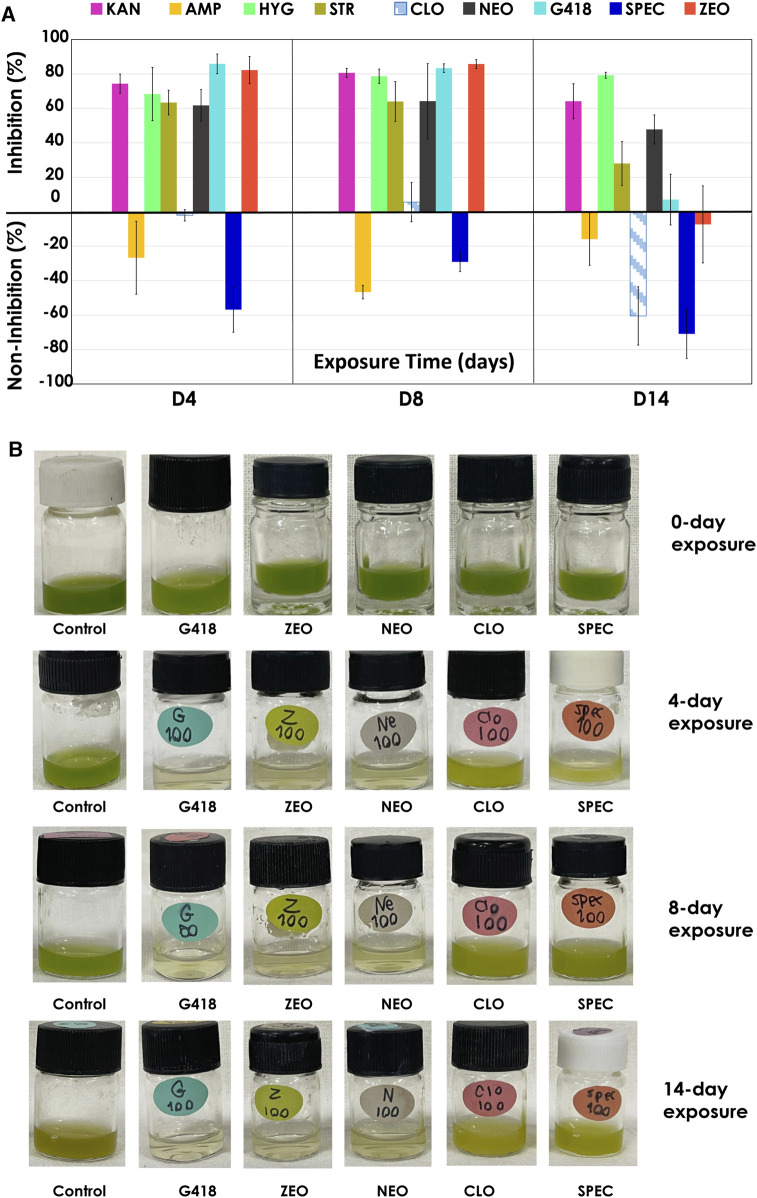
Antibiotics sensitivity of antibiotics under dark mode at 100 μg/mL: **(A)** growth inhibition (%) **(B)** physical characteristic of *C. sorokiniana* AARL G015 of some antibiotics on day 0, day 4, day 8 and day 14.

**FIGURE 5:**
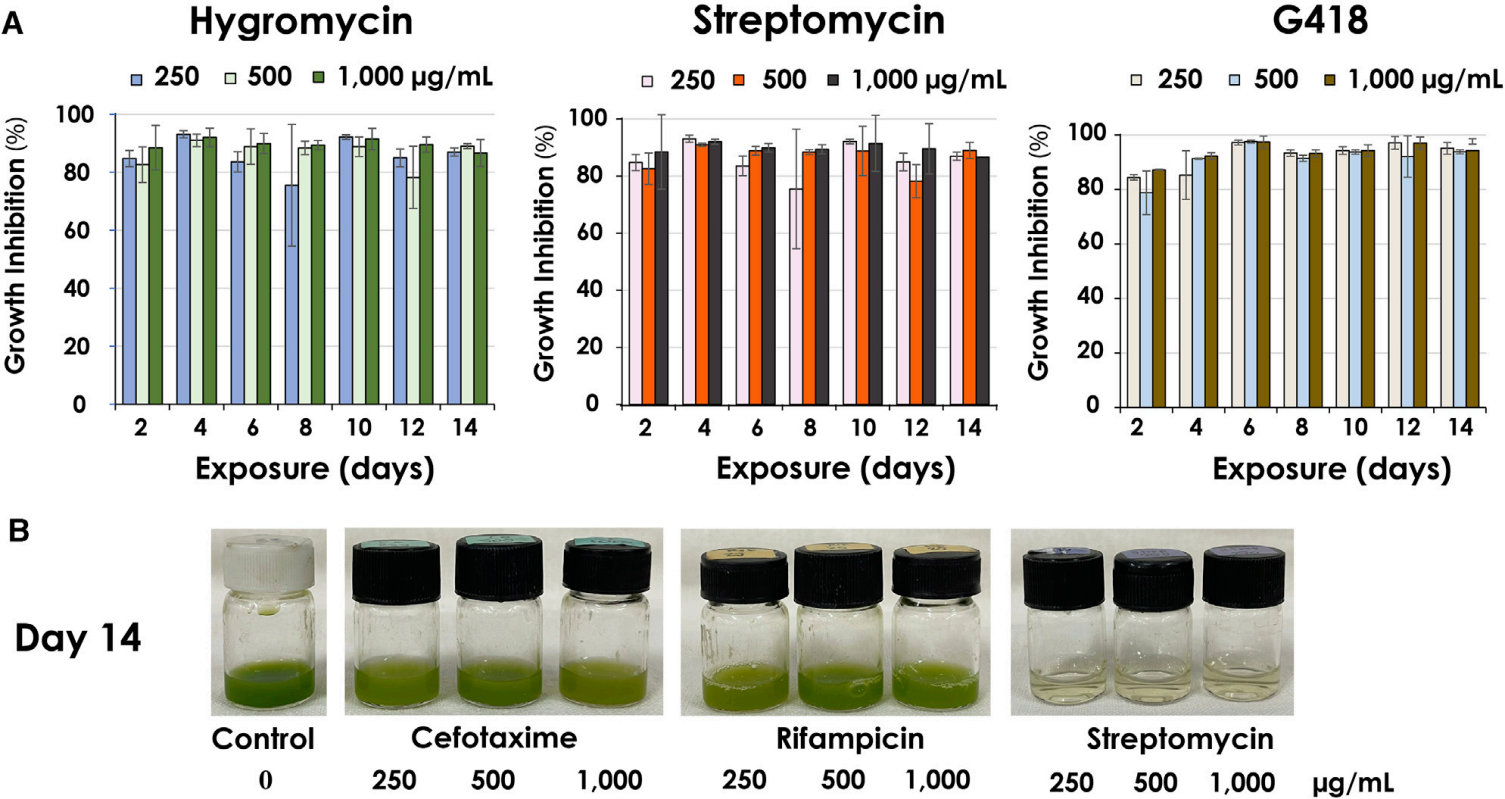
Antibiotics sensitivity at high antibiotic concentrations (250, 500, 1,000 μg/mL) under dark mode of *C. sorokiniana* AARL G015 **(A)** growth inhibition (%) of the most potent antibiotics affecting the microalgal growth after 14 days exposure **(B)** culture color at various antibiotic concentrations on the last day of exposure.

The result from low antibiotic concentrations (25, 50, 100 μg/mL) showed that certain antibiotics could only temporarily inhibit the growth of *C. sorokiniana* AARL G015 during a short exposure period (day 2–4) (Supplementary Figure S1). After this phase, the microalga exhibited a gradual resurgence in growth under dark conditions for STR, CLO, and G418, and NEO (Figure 4A). Intriguingly, the strain became slightly less sensitive to certain antibiotics when exposed to lower concentrations, leading to just slower growth. In particular, SPEC and AMP were unable to inhibit growth entirely. Therefore, the inhibition percentage appeared as negative in Figure 4A, located within the inverted column. However, by the 14-day of exposure, the inhibitory effect of antibiotics dramatically dropped, indicating that this strain is capable of tolerating low antibiotic concentrations, particularly at a concentration of 100 μg/mL of CLO, SPEC, and ZEO, which were unable to inhibit microalgal growth on day 14. Notably, this strain exhibited complete tolerance to AMP at all lower concentrations without decreasing its growth. The physiological changes indicated by the green color in response to low antibiotic concentrations (as shown in Figure 4B) suggested the microalga can survive and gradually grow. Conversely, higher antibiotic concentrations (250, 500, 1,000 μg/mL) caused significant inhibition of the biomass concentration in this strain. *C. sorokiniana* AARL G015 proved to be sensitive to certain antibiotics under dark mode, particularly G418, hygromycin, and streptomycin, which resulted in the highest growth inhibition: 98% at 6 days (500 μg/mL), 93% at 4 days (250 μg/mL), 92% at 4 days (250 μg/mL), respectively (Figure 5A). In contrast, the strain exhibited slightly less sensitivity to AMP and SPEC with growth inhibition of less than 40% at higher concentration (1,000 μg/mL). While CLO demonstrated minimal inhibition at low concentrations, higher concentrations led to the highest growth inhibition (89%) on day 12 (Supplementary Figure S2).

Cefotaxime and rifampicin, commonly used as selection markers for *Agrobacterium*, did not have a strong effect on the microalgal growth even high concentration. As shown in Figure 5B, the physical color of the microalgal culture with CTX and RIF appeared green compared to control culture. Hence, cefotaxime was selected as the suitable marker for *Agrobacterium* in further transformation.

The evaluation and screening of suitable antibiotics become crucial, especially given the lack of research under dark mode for this strain. These results provide essential data for advanced genetic engineering of microalgae and expanding the pool of selectable markers, particularly for heterotrophic cultivation. Currently, for *Chlamydomonas*, a model microalga, six antibiotic resistances are commonly used as selectable markers: zeocin, hygromycin, kanamycin, paromomycin, sulgadiazine, and spectinomycin (De Carpentier et al., 2020). In this study, we found another six antibiotics, namely, G418, HYG, STR, NEO, KAN and CLO, as alternative markers for *C. sorokiniana* AARL G015 under dark cultivation. The suggested antibiotics containing gene cassettes in the plasmid that provide resistance to STR (200 μg/mL), HYG (150 μg/mL), and NEO (100 μg/mL).

### 3.3 *Agrobacterium*-mediated transformation under dark mode

**FIGURE 6:**
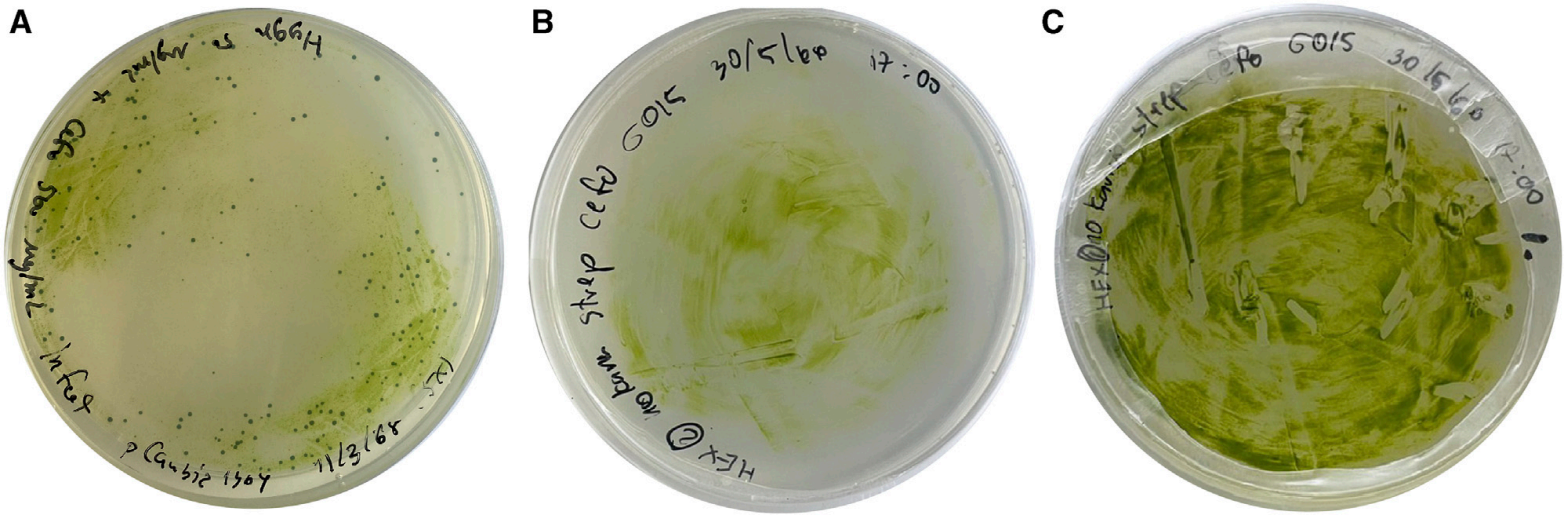
The growth of transformed microalgal cells on solid medium using *Agrobacterium*-mediated transformation method: **(A)** the growth of transformants carrying pCAMBIA 1304 which was contained GFP gene, GUS gene, and HYG gene under selective medium **(B)** the growth of transformants after cocultivation with *Agrobacterium* on day 3, and **(C)** the growth of transformants on selective medium on day 13 at the first round.

The pCAMBIA1304 vector containing GUS gene and *mgfp5* reporter was transformed into *A. tumefaciens* vir helper strain 4404. A single clone of transgenic *A. tumefaciens* was selected for transformation of *C. sorokiniana* AARL G015. Subsequently, the transgenic microalga was selected under agar plate containing STR (200 μg/mL), HYG (150 μg/mL), and NEO (100 μg/mL) as a selectable marker (Figure 6A).

This marks the first study of an efficient genetic transformation system in heterotrophic microalgae, *Chlorella sorokiniana* AARL G015, as a dark host using *Agrobacterium*-mediated transformation to express heterologous genes. *C. sorokiniana* cells were cultured on mBG11 agar medium supplemented with 100 µM of acetosyringone, for 3 days (Figure 6B) at 25°C in complete darkness. The cocultivation process involved the introduction of *Agrobacterium* containing the vector at a cell density of OD_600_ = 0.6. After transformation, the transgenic microalga was fully developed on selective media within 2 weeks (Figure 6C). However, further investigation is required to improve and optimize the transformation efficiency to this heterotrophic strain.

The ability to transform larger-sized genes into the host genome of *A. tumefaciens* has significant advantages on *C. sorokiniana* AARL G015. Previous studies have shown that this transformation method has been successful in a few freshwater microalgal strains, including *Chlamydomonas rienhardii*, *H. pluvialis*, *C. vulgaris*, *Dunaliella tertiolecta* and *C. sorokiniana* (Cha et al., 2012; Sanitha et al., 2014; Norzagaray-Valenzuela et al., 2017; Sharma et al., 2021). Nevertheless, it is important to note that *Agrobacterium*-mediated transformation method has been limited only to auto/mixotrophic cultivation modes. Additionally, various parameters can influence the transformation efficiency using this approach, such as the microalgal species, type of cocultivation media, acetosyringone concentration, mode of microalgal cultivation, and cocultivation duration. Based on the report from Sharma et al. (2021), BG11 cocultivation medium was found to produce the greatest number of transformed colonies for autotrophic *C. sorokiniana* compared to TAP medium, which is commonly used in model microalgae. In this study, mBG11 medium was used for heterotrophic *C. sorokiniana* AARL G015 due to the dark cultivation condition where the microalga needs to use organic carbon sources from medium instead of utilizing energy from light. Moreover, our cocultivation duration was similar to other studies, with resistant colonies of this strain appearing after 3 days of cocultivation (Cha et al., 2012; Sharma et al., 2021).

Another report showed that higher concentrations of acetosynringone than 100 µM can reduce the transformation efficiency (Zho et al., 2009). Therefore, in this study, we used 100 µM as the acetosynringone concentration. There have been research papers discussing genetic approaches to understanding the genes involved in carbon source utilization for enhanced growth, as well as gene regulation to control glucose metabolism. However, there is a lack of information on heterologous genes expression on heterotrophic cultivation. This research demonstrated that certain strains, particularly *C. sorokiniana* AARL G015, which can grow better in heterotrophic mode, should be studied for the development of their genetic transformation under dark cultivation conditions**.**

### 3.4 GUS biochemical expression

**FIGURE 7:**
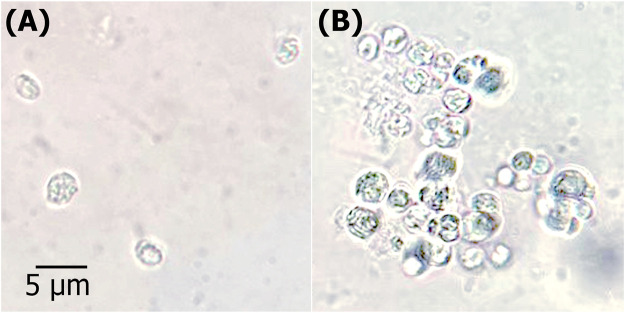
GUS histochemical analysis. Microscopic view of transformation of heterotrophic microalga with pCAMBIA1304 vector harboring GUS gene reporter **(A)** WT; untransformed control *Chlorella sorokiniana* **(B)** T; transgenic *C. sorokiniana* showing GUS expression.

GUS histochemical assay was performed to check GUS expression as a reporter in transformed cells, following the antibiotic screening for resistant cells. This assay allowed transformants to react with the substrate (X-gluc), resulting in blue color appearance. Figure 7 shows the transformed *C. sorokiniana* AARL G015 in weakly blue-colored cells, while the non-transformant shows no blue color. However, the screening for transient GUS expression exhibited weak coloration. There are many variables that affect the quality of the histochemical localization. Several factors are involved in the GUS gene expression pattern, such as promoter efficiency, random positioning effects in the nuclear genome, the rigid cell wall, which may possibly lead to improper penetration of GUS substrate, and a lack of GUS gene expression, as well as epigenetic-derived transgene silencing (Kathiresan et al., 2009; Doron et al., 2016; Norzagaray-Valenzuela et al., 2017; Miamioh, 2019). As a consequence, the transgenic cells were visualized using GFP gene as a reporter.

Hence, there is a need to study and improve this technique for many other microalgal species. Using the GUS gene as a reporter makes transgenic monitoring easier and less time-consuming (Su et al., 2016). Additionally, further studies are necessary to explore factors that may influence the expression of GUS gene in specific strains. The goal is to enhance GUS gene expression and address gaps in the genetic toolbox for non-model microalgae.

### 3.5 Detection of GFP in transformed algal cells

**FIGURE 8:**
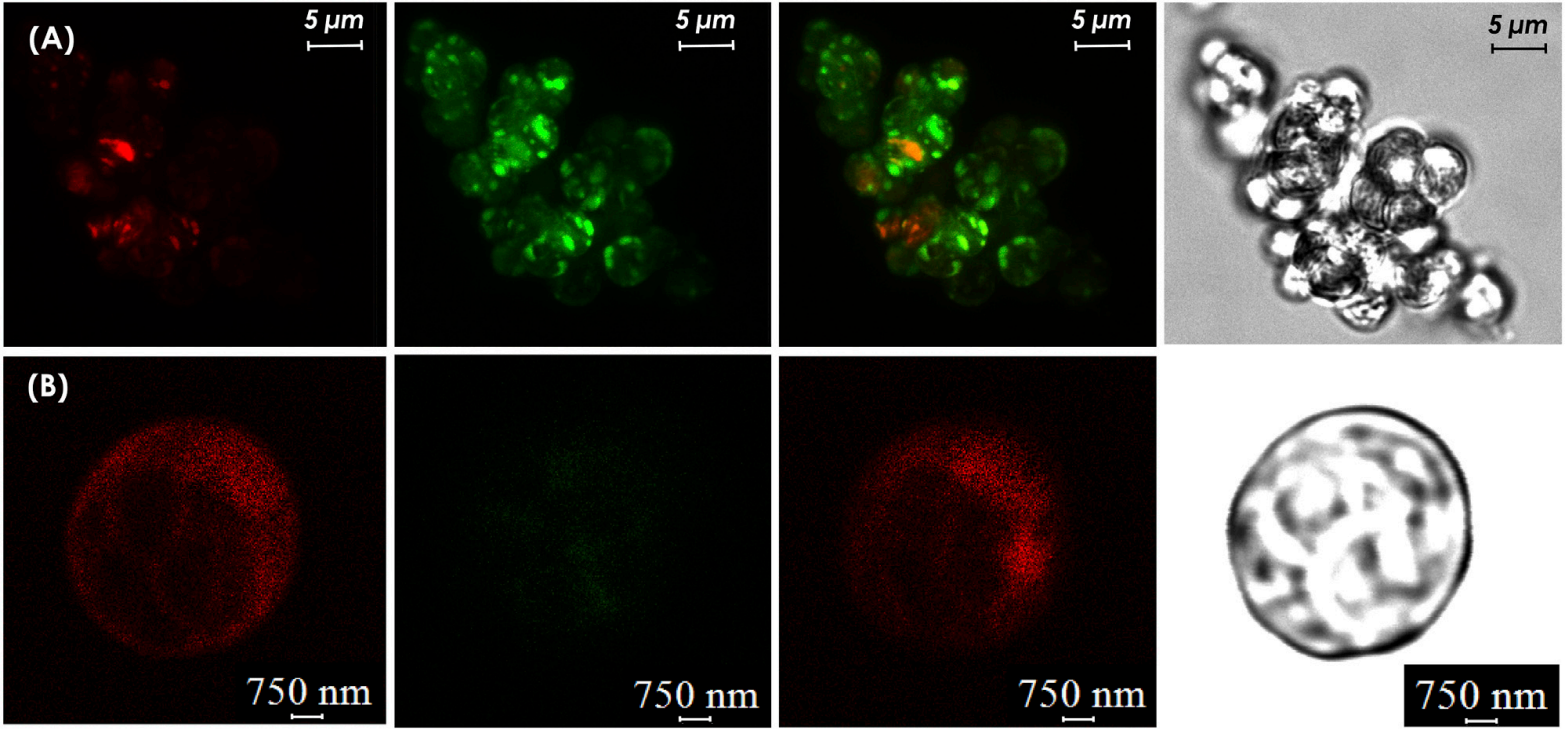
Confocal microscopy analysis **(A)** GFP expression in transgenic cell culture **(B)** wild-type cell cultures. From left to right: (i) cells detection in chloroplast auto-fluorescence channel, (ii) cells detection in the GFP fluorescence channel, (iii) merged images, and (iv) phase contrast image cells.

Visualization of GFP using a laser confocal microscopy (Leica stellaris 5, Germany) confirmed the expression of GFP in transgenic cells as green, while non-transformed cells exhibited only red chlorophyll autofluorescence. The putative *C. sorokiniana* transformants carrying the pCAMBIA1304 vector were recovered for the third time in liquid-selection medium. The result demonstrated that transgenic *C. sorokiniana* containing pCAMBIA1304 could be easily distinguished from non-transformant cells (Figure 8). Strong fluorescence emissions were observed in *C. sorokiniana* with pCAMBIA1304 and were localized in the cells where the bright green signal appeared, whereas non-transformants showed no green.

### 3.6 Stability of transformants

After undergoing 5-7 rounds of subcultures on a non-selective medium, followed by transferring to the specific selective medium, *Chlorella* transformants were still able to grow on the selective medium. This outcome implies the stability of transgenes within *C. sorokiniana* AAGL G015. In another study, the use of *Agrobacterium* transformation for delivery of exogenous materials to *Euglena gracilis* demonstrated the stability of the mutants for over a year, through 12 rounds of cultivation. In contrast, other transformation methods such as biolistic bombardment, and electroporation techniques lacked stable integration of the transforming DNA into the host genome (Chen et al., 2022).

Have hypes and hopes by using this advancement technologies in dark-microalgal host? Tremendous breakthroughs in the new discovery of novel expression platforms for producing biopharmaceuticals or phytochemicals are needed. Heterotrophic microalgae, as a sustainable and scalable host for recombinant technology, hold promise. Certain microalgal species naturally possess pathways to synthesize vital substances for nutrition and food. Recent progress in microalgae-based product development has yielded various industrial applications, including enhanced textural and color properties, improved sensory quality, antioxidant capabilities, and elevated protein content. Additionally, microalgae represent a third-generation biofuel and energy source, benefitting from their short life cycle, environmental adaptability, and widespread distribution that align well with economic systems. However, for reasons of high cost and unavailability of genetic information for commercially suitable strains, they have not yet reached industrial maturity and commercial success. So far, a considerable effort has been given to tackle the bottleneck of various methods, including various nutritional-, environmental-, and physiological alteration of cultivation, metabolic and genetic engineering (Pierobon et al., 2018; Chen and Lee, 2019). To satisfy the large market demand, a high level of technology and mechanized harvesting techniques are required. Future studies must explore the integration of new efficient technology of downstream processes including extraction, concentration, conversion, and purification of recombinant product from microalgae. It is also essential to establish microalgal host using genetically modified microalga for both endogenous and heterologous expression.

To achieve economically efficient large-scale production and utilization of microalgae for production, sustainable and eco-innovative processing techniques are necessary. These techniques should efficiently transform raw microalgal biomass into value-added products or isolated ingredients without compromising on their nutritional and environmental benefits. Using microalgal hosts to produce plant substances offers advantages such as consistent synthesis without seasonal limitations and serves as a valuable tool for studying the involved precursors and enzymes of desired phytochemicals (Koo et al., 2013; Eisohly et al., 2014). Choosing a suitable host for plant production requires careful consideration, especially with the projected increase in global demand for production along with zero waste. This marks a new phase for the alternative resources and tools, fostering innovation in this sector.

Additionally, the ability to manipulate microalgae’s excretion system could open up possibilities for direct release of fuels or other metabolites into the medium, enhancing their potential as versatile bioproduction platforms. Microalgae also show promise in producing recombinant proteins of high industrial relevance due to their rapid growth cycle and cost-effective cultivation compared to other expression hosts (Gramegna et al., 2020). These advancements shed light on the molecular aspects of algal phytochemical production and pave the way for optimizing microalgae as a platform for therapeutic and industrially significant recombinant protein production. As a ‘green’ alternative to existing mammalian, yeast, or bacterial systems, microalgae are poised to play a prominent role in the future of biotechnology and functional product industries.

## 4 Conclusion

Our work presented the first report on the successful transformation of the green microalga, *C. sorokiniana* AARL G015, using *Agrobacterium*-mediated transformation under heterotrophic cultivation. The transformation was confirmed through GUS and GFP expression analysis. To enhance the transformation efficiency, further additional optimization of specific strains and cultivation modes should be evaluated. The successful transformation of heterologous genes through a cost-effective technique under completely dark mode marked a significant advancement in the field of engineered microalgae. This finding provides a molecular toolkit for the high-value production of phytochemicals for further industrial applications, offering a cost-effective, less labor-intensive, and time-saving alternative platform. Furthermore, the development of new molecular tools and techniques is crucial for fully harnessing the economic potential of microalgae as circular model organisms. Genetic engineering improvement of specific strains and exploration of promising hosts for downstream applications are valuable areas for future research. This research marks a prominent step and fill the gap towards unlocking the potential of heterotrophic microalgae for genetic engineering applications. Further development of methods and strategies for transgene expression in non-model microalgae are critically required.

## Data Availability

The original contributions presented in the study are included in the article/Supplementary Material, further inquiries can be directed to the corresponding author.
