# Supplementary material for: Unlocking microalgal host—exploring dark-growing microalgae transformation for sustainable high-value phytochemical production
Source: Front Bioeng Biotechnol. 2023 Nov 9;11:1296216. doi: 10.3389/fbioe.2023.1296216 (PMC10666632; doi:10.3389/fbioe.2023.1296216)
Supplement: Supplementary file 1 [file DataSheet1.docx]

Supplementary Material

Unlocking Microalgal Host – Exploring Dark-growing Microalgae Transformation for Sustainable High-Value Phytochemical Production

Surumpa Jareonsin*, Kanjana Mahanil, Kittiya Phinyo, Sirasit Srinuanpan, Jeeraporn Pekkoh, Masafumi Kameya, Hiroyuki Arai, Masaharu Ishii, Ruttaporn Chundet, Pachara Sattayawat, Chayakorn Pumas

*** Correspondence:** Corresponding Author: chayakorn.pumas@gmail.com

# Supplementary Figures and Tables

## Supplementary Figures


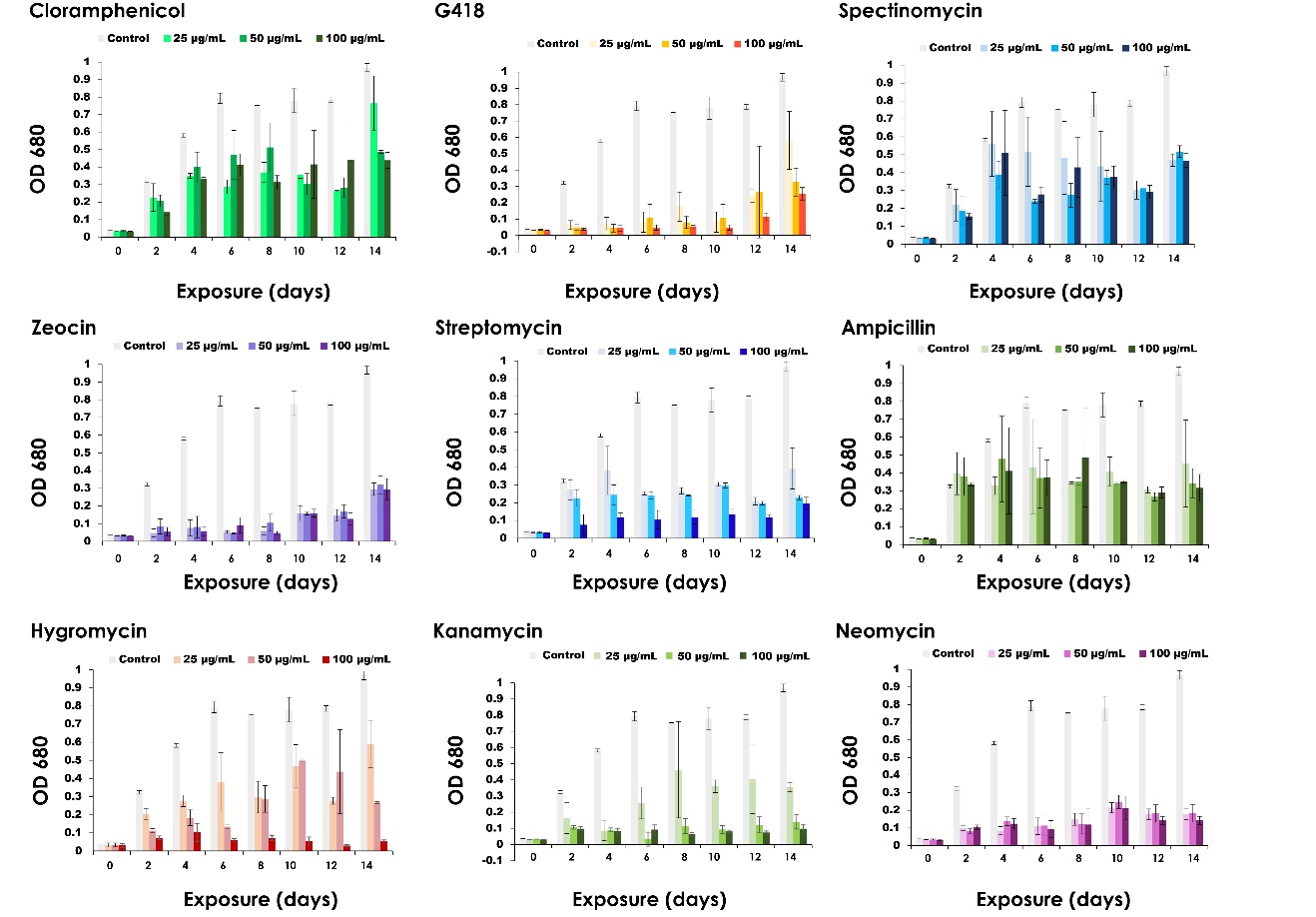


**Supplementary Figure 1.** *Chlorella sorokiniana* AARL G015 growth curve (optical density; OD 680) under lower antibiotic concentrations (25, 50, 100 µg/mL) in the dark cultivation during 14 days of exposure


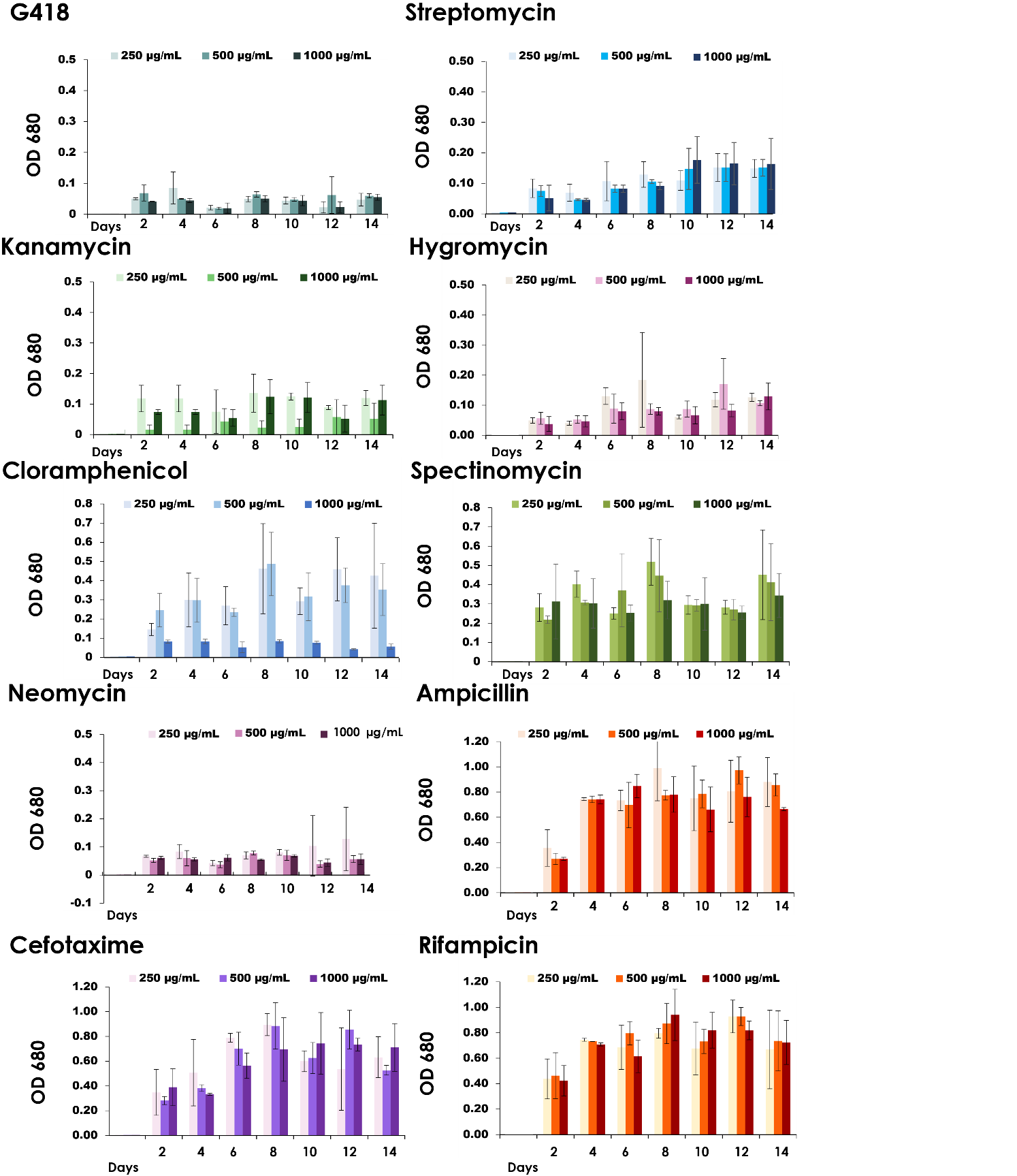


**Supplementary Figure 2.** *Chlorella sorokiniana* AARL G015 growth curve (optical density; OD 680) under higher antibiotic concentrations (250, 500, 1,000 µg/mL) in the dark cultivation during 14 days of exposure
